# Characterization of a Bifunctional *O*- and *N*-Glucosyltransferase from *Vitis vinifera* in Glucosylating Phenolic Compounds and 3,4-dichloroaniline in *Pichia pastoris* and *Arabidopsis thaliana*

**DOI:** 10.1371/journal.pone.0080449

**Published:** 2013-11-14

**Authors:** Zhi-Sheng Xu, Wei Xue, Ai-Sheng Xiong, Ya-Qiu Lin, Jing Xu, Bo Zhu, Wei Zhao, Ri-He Peng, Quan-Hong Yao

**Affiliations:** 1 Agro-Biotechnology Research Center, Shanghai Academy of Agricultural Sciences, Shanghai, China; 2 College of Horticulture, Nanjing Agricultural University, Nanjing, Jiangsu, China; 3 College of Fisheries and Life Science, Shanghai Ocean University, Shanghai, China; Florida International University, United States of America

## Abstract

2,4,5-Trichlorophenol, 2,6-dimethylphenol, 3-methylcatechol, phenol, hydroquinone, catechol, and 3,4-dichloroaniline are present in the environment and are risky to humans and animals because of their wide applications in many industries. In this study, a putative uridine diphosphate glucose-dependent glycosyltransferase from *Vitis vinifera* (VvUGT72B1) displayed high *O*-glucosyltransferase or *N*-glucosyltransferase activity toward all these xenbiotics and was able to enhance the resistance of *P. pastoris* to them. Compared with wild-type *Arabidopsis* plants, VvUGT72B1-transgenic *Arabidopsis* plants showed higher resistance to all the xenobiotics except for phenol and exhibited higher removal efficiencies against all xenobiotics. Glucosides of 3-methylcatechol, 2,6-dimethylphenol, phenol, and 3,4-dichloroaniline were exported to the surrounding media by *Arabidopsis* plants while transgenic *Arabidopsis* plants exported more glucosides than wild-type *Arabidopsis* plants. Our findings have the potential to provide a broader spectrum remediation strategy for the phytoremoval and degradation of phenolic compounds and 3,4-dichloroaniline than previous works.

## Introduction

2,4,5-Trichlorophenol (TCP) and 3,4-dichloroaniline (DCA) are both synthetic intermediates and degradation products of a number of pesticides [1–3]. 2,4,5-TCP induces oxidative damage in human erythrocytes [4]. Phenol and 2,6-dimethylphenol (DMP) are listed as a priority pollutant by the US Environmental Protection Agency [5]. 3-Methylcatechol (MC) is a pollutant and a metabolite of toluene, which is cytotoxic to humans and rats [6]. Rats can be induced to produce renal tubule adenomas and to exacerbate spontaneous chronic progressive nephropathy by hydroquinone [7]. However, arbutin, an inhibitor of the biosynthesis of the human pigment melanin, is an O-β-d -glucoside of hydroquinone [8]. Catechol can cause eczematous dermatitis in humans, depression of the central nervous system, and prolonged rise in blood pressure in animals [9]. These xenobiotics and their derivatives are introduced into the environment by human activities because of their extensive applications.

Plants are widely used in remediation studies because of their various advantages [10–14]. Plants transform xenobiotics with a three-phase detoxification system: conversion, conjugation, and compartmentalization [10]. Glycosylation is the most commonly observed conjugation reaction in plants. In this process, the conjugation of the activated xenobiotic with sugar is catalyzed by glycosyltransferases (GTs) such as uridine diphosphate glucose-dependent glycosyltransferases (UGTs) [15–17]. AtUGT72B1 showed the highest UGT activity toward 2,4,5-TCP and 3,4-DCA among all UGTs in *A. thaliana* [18]. PtUGT72B1 displayed higher TCP-conjugating activity than AtUGT72B1 and also showed high glucosylation ability toward phenol, hdroquinone and catechol [13,14].

In this paper, we analyzed a novel GT72B1-like glycosyltransferase (GenBank Accession No: XP_002280923) from *Vitis vinifera* to obtain its functional information. Our goals are to determine whether this novel glycosyltransferase can glucosylate more kinds of phenolic compounds (PCs) than PtUGT72B1 and has *N*- glycosyltransferase avtivity toward 3,4-DCA like AtUGT72B1 and ultimately enhance a plant’s ability to detoxify and remedy these xenobiotics.

## Materials and Methods

### Design and chemical synthesis of *VvUGT72B1* gene

According to the amino acid sequence (GenBank Accession No: XP_002280923) of the putative hydroquinone glycosyltransferase gene *VvUGT72B1* from *V. vinifera*, this gene was synthesized by a PCR-based two-step DNA synthesis method [19,20]. *VvUGT72B1* gene was modified using a previously described method [13].

### *VvUGT72B1* gene expression in *P. pastoris*

The synthesized *VvUGT72B1* gene was transformed into *P. pastoris* strain GS115 cells by electroporation as previously described [21]. The electroporated cells were plated on histidine-deficient SD medium and incubated at 30 °C for 3 days. All transoformants were pooled and resuspended in distilled water. Cell density was determined using a hemocytometer, and cells were plated at 5 × 10^5^ cells per standard 8.5 cm Petri dish on YPD agar medium containing 0.4 mg/mL G418. To choose the positive clones expressing VvUGT72B1, G418-resistant transformants were cultured in BMGY medium for 2 days and subsequently transferred to BMMY medium for 1 day to induce expression with constant shaking at 200 rpm and 30 °C as previously described [13]. Yeast protein was extracted and determined by polyacrilamide gel electrophoresis (SDS-PAGE). Every band was quantified with a Shine Tech Gel Analyzer (Shanghai Shine Science of Technology Co., Ltd., China) as previously described [13]. Protein from *P. pastoris* containing empty pPIC9K vector was also extracted and used as a control.

### Generation of transgenic plants with the synthesized *VvUGT72B1* gene

The synthesized *VvUGT72B1* gene was transformed into *A. thaliana* (ecotype Colubia) plants with the hygromycin B phosphotransferase gene as the genetic selection marker under the control of CaMV 35S promoter and ocs terminator based on a previous study [13]. T_3_ homozygous transgenic lines selected on Murashige and Skoog (MS) [22] medium supplemented with hygromycin (50 mg/L) were used for all subsequent analyses. Wild-type (WT) *A. thaliana* was used as control.

### Reverse transcription PCR analysis of *A. thaliana* plants

The extraction of total RNA from *A. thaliana* plants and the synthesis of first-strand cDNA were performed as previously described [13]. The fragment of *VvUGT72B1* gene was amplified by PCR using a forward primer 5'-ACGACGCTTACAAGTGGGTT-3' and a reverse primer 5'-AGTGGCCATGCGATCATAGG-3'. The *A. thaliana* actin gene (*AtAc*2; accession number: NM112764) was used as an internal standard. The fragment of the actin gene was amplified with the primers as previously described [13]. Amplification was performed at 94 °C for 10 min for the first cycle and then 27 cycles of 94 °C for 30 s, 56 °C for 30 s, 72 °C for 45 s, followed by an additional 5 min at 72 °C. The PCR products were separated on 1.2% agarose gel and quantified using a Model Gel Doc 1000 (Bio-Rad, USA). The DNA intensity ratio of *VvUGT72B1* gene to *AtAc2* was determined with a Shine Tech Gel Analyzer (Shanghai Shine Science of Technology Co., Ltd., China) to evaluate the expression pattern of *VvUGT72B1* gene.

### Extraction and activity assay of VvUGT72B1 enzyme

Protein was extracted from *P. pastoris* cells and was stored in buffer at -20 °C prior to testing its UGT activity as previously described [14]. 3,4-DCA and six kinds of PCs, including 2,4,5-TCP, 2,6-DMP, phenol, hydroquinone, catechol, and 3-MC, were used in this study. UGT activity assay was performed by using previously described method [14]. The reaction was carried out at 30 °C for 4 h. After incubation, the reaction was stopped by adding 200 μL of ethanol, followed by vortexing (60 s) and centrifugation (20 000 × *g*, 10 min). The supernatant was used for reverse-phase HPLC analysis. All data were determined and averaged from three independent assays.

Extraction and quantification of *Arabidopsis* crude protein were performed using previously described methods [14]. UGT activity assay was performed as described above.

### *P. pastoris* and plant resistance assay

To assay yeast resistance, *P. pastoris* strain GS115 transformants (transfected with linearized pPIC9K or pPIC-VvUGT72B1) were grown in BMGY medium. After induction in BMMY medium for 24 h, yeast cells were diluted to 5 × 10^3^ cells/µL. About 5 µL of cells were spotted on fresh BMMY agar plates containing 200 µL of methanol (as control), 0.46 mM 3,4-DCA, 45.58 µM 2,4,5-TCP, 4.09 mM 2,6-DMP, 8.50 mM phenol, 0.91 mM hydroquinone, 6.36 mM catechol, or 1.21 mM 3-MC (predissolved in methanol) and then incubated at 30 °C for 2 days.

To test plant resistance, about 30 seeds of WT or VvUGT21B1-transgenic (VT) *A. thaliana* plants were germinated on MS agar plates containing 20 µL of ethanol (as control), 0.25 mM 3,4-DCA, 30.39 µM 2,4,5-TCP, 0.20 mM 2,6-DMP, 0.37 mM phenol, 0.45 mM hydroquinone, 0.18 mM catechol, or 0.40 mM 3-MC (predissolved in ethanol). Plants were grown vertically for 10 days. Each treatment was performed thrice.

### Feeding studies with 3,4-DCA and PCs

Twenty 1-week-old WT or VT seedlings were transferred to 50 mL flasks containing 20 mL of liquid MS medium at 24 °C in 16/8 h light on an orbital shaker (60 rpm). Seven days later, the liquid media were replenished with 20 mL of new MS liquid medium containing 0.62 mM 3,4-DCA, 50.65 µM 2,4,5-TCP, 0.82 mM 2,6-DMP, 1.06 mM phenol, 1.82 mM hydroquinone, 1.73 mM catechol, or 1.21 mM 3-MC (predissolved in ethanol). The concentrations of PCs and their glucosides in growth medium were determined every 24 h by reverse-phase HPLC. For glucosides that were not available, their relative content in media were presented as the area of the peak obtained during HPLC analysis (mAU·s). Media added with xenobiotic without plants were used as control. The content of glucosides in the plants was determined by using method previously described [14].

### Measurements of PCs and their glucosides by HPLC and LC-MS

Standards of arbutin, β-d-glucopyranoside (PGPS), 3,4-DCA and all PCs used in this study were purchased from Sigma. Reverse-phase HPLC was performed using an Agilent 1100 HPLC system (Agilent Technologies, CA, USA) and a Columbus 5 µm C18 column (250 mm × 4.60 mm; Phenomenex). Glucosides of 3,4-DCA, 2,4,5-TCP, 2,6-DMP, and 3-MC were separated from their aglycones by a linear gradient of 20% to 70% acetonitrile in H2O for 20 min at 213 nm. Phenol and catechol were separated from their glucosides by a linear gradient of 10% to 30% acetonitrile in H2O for 10 min. For hydroquinone, a linear gradient of 5% to 10% acetonitrile in H2O for 10 min was used as the mobile phase. Since the glucosides of 3,4-DCA, 2,6-DMP, and 3-MC were not available, liquid chromatography-mass spectrometry (LC-MS) analyses were performed to determine the products. 20 μL of the whole reaction mixture was applied to LC-MS. Glucosides of 3,4-DCA, 2,6-DMP, and 3-MC were separated from their aglycones by a linear gradient of 20% to 70% acetonitrile in H_2_O at 0.5 mL/min for 20 min and monitored at 213 nm. The peak corresponding to the product was subjected to mass spectrometry analysis. Positive ion mass spectrometry analyses were performed for glucosides of 2,6-DMP, and 3-MC with electrospray ionization source on 3849 V, 10.0 L/min drying gas flow, 30 psi nebulizer pressure, and 350 °C drying gas temperature. For glucosides of 3,4-DCA, negative ion mass spectrometry analysis was performed.

## Results

### Synthesis of *VvUGT72B1* gene from *V. vinifera*

We synthesized the putative glycosyltransferase gene *VvUGT72B1* based on the encoding amino acid of the WT gene from *V. vinifera* (GenBank Accession No: XP_002280923). BLAST search showed that the synthesized gene was 79.03% identical to the WT gene. The A+T content of the WT gene was 46.5%, whereas the A+T content of the synthesized gene was 49.9% because of the modification (Figure S1).

### Structure analysis of VvUGT72B1

**Figure 1:**
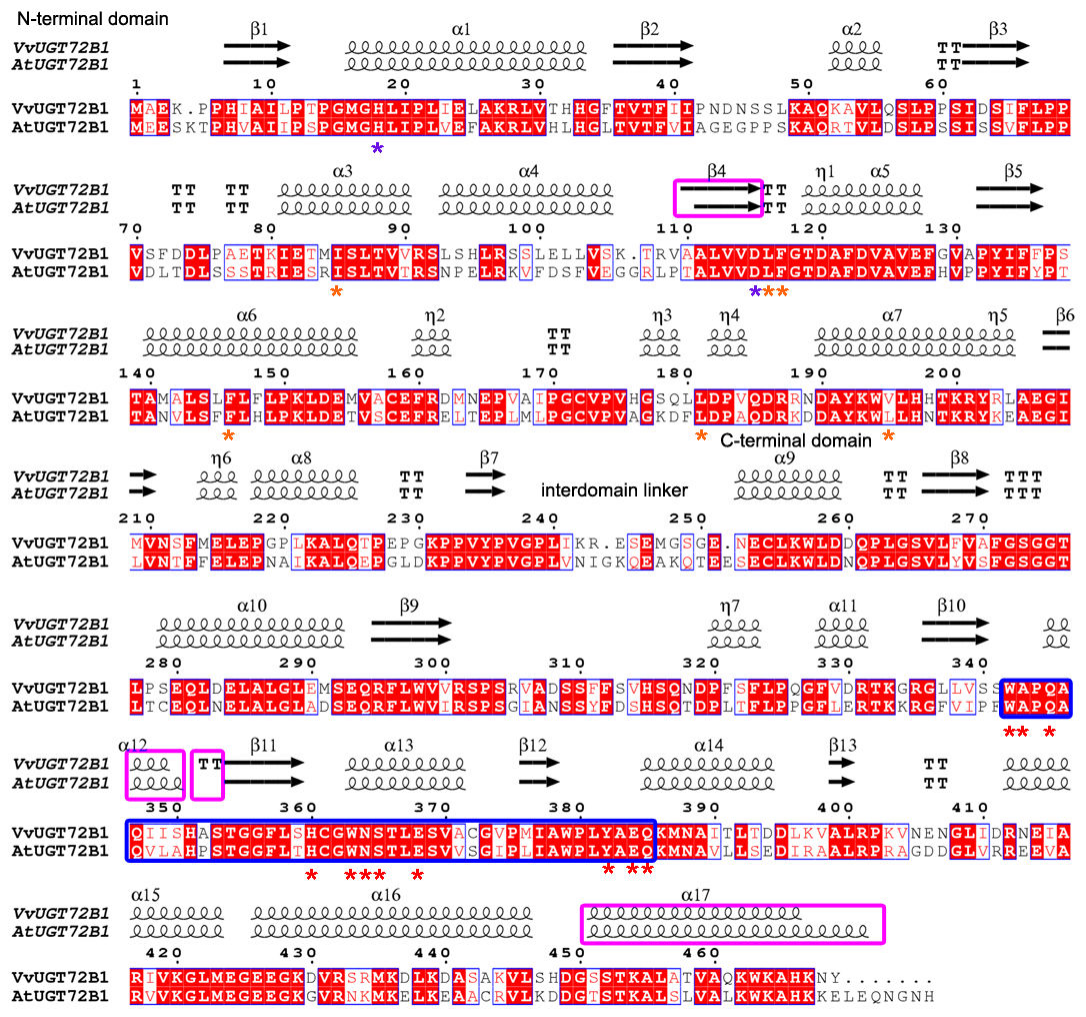
Structure-based sequence alignment of VvUGT72B1 and AtUGT72B1. Secondary structure elements are shown above the alignment. Conserved residues are highlighted. The UGT signature PSPG motifs are enclosed in a bold blue box. Conserved residues involved in sugar donor binding are indicated with a red asterisk (*) below the alignment. Conserved residues interacting with the acceptor are indicated with a purple asterisk. Residues involved in enclosing TCP in AtUGT72B1 are indicated with a saffron yellow asterisk. “α” represents an α-helix, “β” represents a β-sheet, “TT” represents a β-turn, and “η” represents 3.10 helix. This figure was produced using ENDscript [31].

Structural comparison was performed between VvUGT72B1 and AtUGT72B1. Secondary structure analysis revealed that VvUGT72B1 was similar to AtUGT72B1 (Figure 1), except for the β4-sheet, α12-helix, β-turn between α12-helix and β11-sheet, as well as α17-helix (in pink boxes). The overall tertiary structure of VvUGT72B1 was similar to that of AtUGT72B1, displaying a similar GT-B fold (Figure S2A). Although, amino acid sequence identity between these two plant UGTs was only about 65%. VvUGT72B1 (469 amino acid residues) was shorter with 11 less residues than AtUGT72B1 (480 amino acid residues).

VvUGT72B1 consisted of N- and C-terminal domains that were very tightly packed with one another. The UGT signature plant secondary product glycosyltransferase (PSPG) motif, with most of the key residues conserved, existed in the C-terminal domain of VvUGT72B1. The pocket consisting of these key residues of VvUGT72B1 was similar to the sugar donor-binding pockets identified in the structures of AtUGT72B1, MtUGT85H2, MtUGT71G1, and VvGT1 [18,23–26]. VvUGT72B1 may interact with UDP-glucose mainly through these residues in the UGT signature PSPG motif (Figure S2B). Cys361 and Trp360 in MtUGT85H2, Ala340 and Trp339 in MtUGT71G1, as well as Trp332 in VvGT1 were in contact with the uracil ring of the UDP-sugar donor [23,24,26]. In VvUGT72B1, the corresponding residues were Ala343 and Trp342 (Ala347 and Trp346 in AtUGT72B1). Gln335 and Glu358 in VvGT1, Gln363 and Glu386 in MtUGT85H2, as well as Gln342 and Glu365 in MtUGT71G1 formed hydrogen bonds with the ribose of the UDP-sugar donor [24–26]. In VvUGT72B1, the corresponding residues were Gln345 and Glu368 (corresponding to Gln349 and Glu372 in AtUGT72B1). Asn382, Ser383, and His378 in MtUGT85H2, Asn361, Ser362, and His357 in MtUGT71G1, as well as Ser355 in VvGT1 interacted with the α-phosphate group of UDP-sugar [24–26]. The equivalents in VvUGT72B1 were Asn364, Ser365, and His360 in VvUGT72B1 (Asn368, Ser369, and His364 in AtUGT72B1). His378 in MtUGT85H2, Tyr379 and His357 in MtUGT71G1, as well as His350 in VvGT1 interacted with the β-phosphate group of the donor molecule [24–26]. The equivalents in VvUGT72B1 were Tyr382 and His360 (corresponding to Tyr386 and His364 in AtUGT72B1). Trp360, Glu381, and Gln382 in MtUGT71G1, as well as Trp353, Glu374, and Gln375 in VvGT1 provided structural support for the binding sugar group of the donor molecule [24,26]. The equivalents in VvUGT72B1 were Trp363, Glu384, and Gln385 (corresponding to Trp367, Glu388, and Gln389 in AtUGT72B1).

A narrow, deep pocket consisting of residues from the N-terminal domain was similar to that of acceptor binding sites in MtUGT71G1, AtUGT72B1, and VvGT1. 2,4,5-TCP enclosed by Glu83, Ile86, Leu118, Phe119, Phe148, Leu183, and Leu197 were identified in AtUGT72B1 [18]. In VvUGT72B1, these corresponding residues were Glu82, Ile85, Leu116, Phe117, Phe146, Leu116, and Val195, respectively. His19 and Asp117 in AtUGT72B1, His22 and Asp121 in MtUGT71G1, His20 and Asp119 in VvGT1, as well as His21 and Asp 125 in MtUGT85H2 were proven to be involved in the deprotonation of acceptor molecules [18,23–26]. The corresponding residues in VvUGT72B1 were His18 and Asp115. The similarities between VvUGT72B1 and AtUGT72B1 suggested that VvUGT72B1 may use UDP-glucose as a donor and be able to glucosylate the same substrates as AtUGT72B1, such as 3,4-DCA and 2,4,5-TCP.

### Expression of VvUGT72B1 in *P. pastoris*

**Figure 2:**
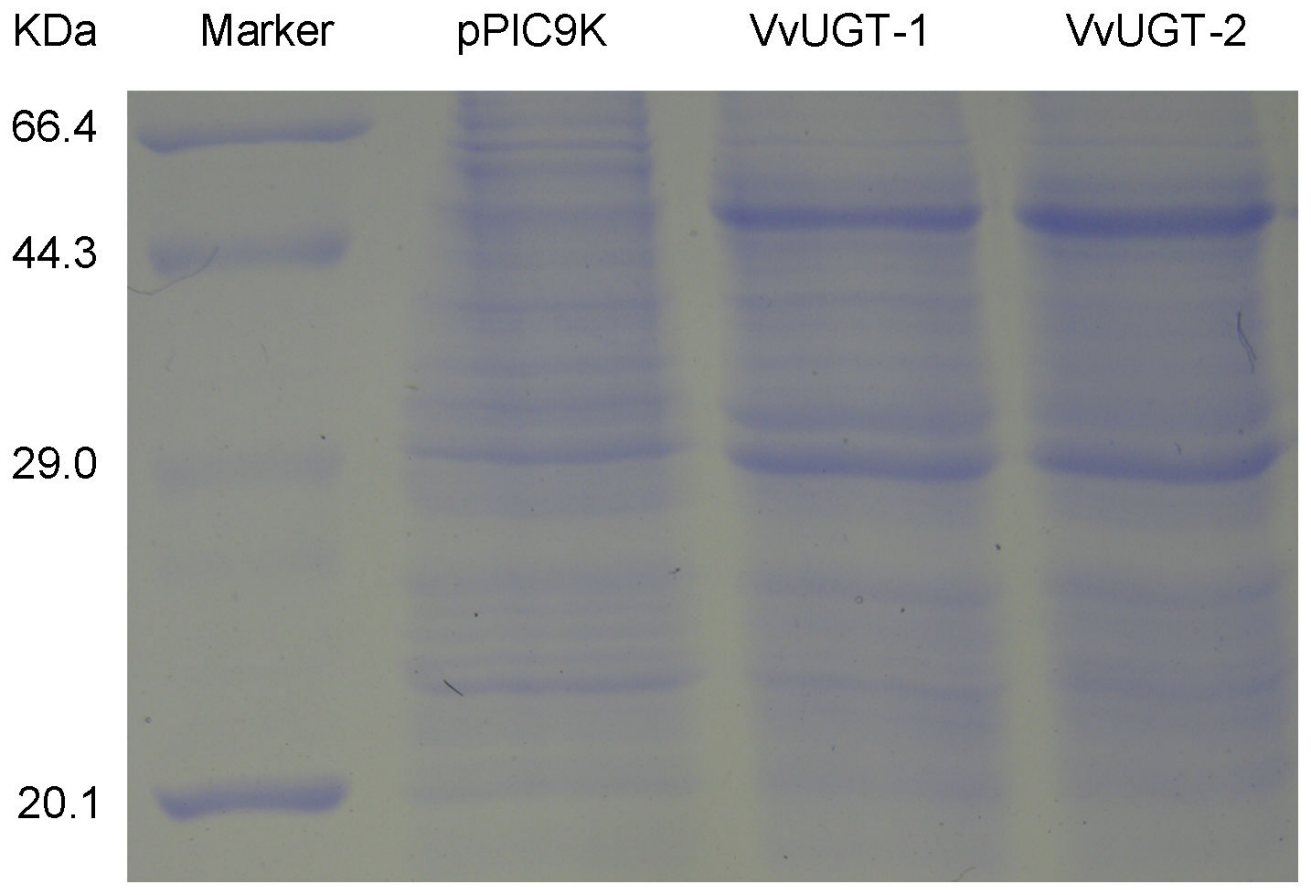
Expression of the putative glycosyltransferase gene *VvUGT72B1* in *Pichia pastoris*. UGT was analyzed on a 12% (w/v) polyacrylamide gel and visualized with Coomassie Brilliant Blue staining.

After two steps of selections on histidine-deficient SD and YPD media containing 400 mg/L G418, 20 colonies were selected for further induction in BMMY to screen the expression strain by SDS-PAGE. Nine strains expressed the recombinant VvUGT72B1. The recombinant VvUGT72B1 was about 53 kDa (Figure 2). This 53-kDa polypeptide corresponding to recombinant VvUGT72B1 was absent in the crude protein extracts from *P. pastoris* containing empty pPIC9K vector. The content of recombinant VvUGT72B1 reached almost 50% of the total protein after induction for 24 h.

### UGT activity of VvUGT72B1 *in vitro*

**Figure 3:**
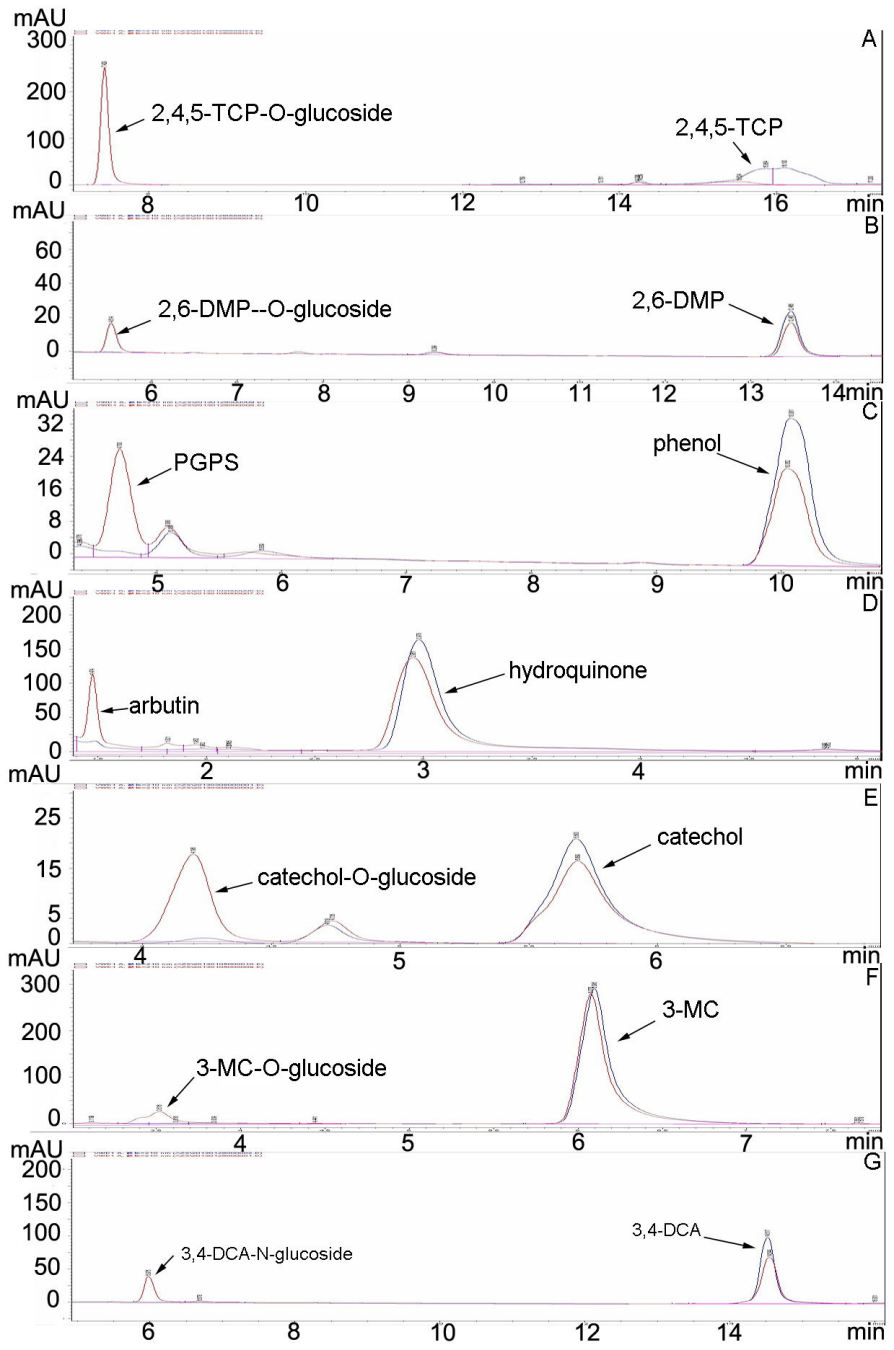
Yeast cells’ resistances to xenbiotics. The resistance of *P. pastoris* transformants expressing VvUGT72B1 or containing empty vector pPIC9K to methanol, 2,4,5-TCP, 2,6-DMP, phenol, hydroquinone, catechol, 3-MC, and 3,4-DCA.

To investigate the ability of VvUGT72B1 for glucosylating 3,4-DCA and PCs, crude enzyme extracts from *P. pastoris* expressing recombinant VvUGT72B1 were examined. The structures of the substrates tested are shown in Figure S3. Crude protein extract from *P. pastoris* containing empty pPIC9K vector was used as a control. The crude enzyme extracts containing recombinant VvUGT72B1 more quickly consumed 3,4-DCA and PCs than the control after adding UDP-glucose (Figure 3). As expected, new peaks in HPLC analyses corresponding to putative glucosides of 3,4-DCA and PCs were observed after incubation. These new peaks were absent in control experiments, except for catechol. The putative glucosides were also absent after crude enzyme extracts containing recombinant VvUGT72B1 were incubated with 3,4-DCA, PCs, or UDPG (data not shown). The putative glucosides of 2,4,5-TCP, phenol, and hydroquinone were confirmed to be 2,4,5-TCP-1-O-glucoside, phenyl-β-d-glucopyranoside (1-O-glucoside of phenol), and arbutin(1-O-glucoside of hydroquinone), respectively, as determined by comparing their retention time in HPLC analyses with previously described results [13,14]. The putative glucoside of catechol was confirmed to be catechol-1-O-glucoside by comparing its retention time under the same HPLC analysis condition with PtUGT72B1-conjugated catechol (data not shown), which was confirmed to be catechol-1-O-glucoside by mass spectrometry [14]. To identify the masses corresponding to the peaks of putative 3,4-DCA-glucoside, 2,6-DMP-glucoside, and 3-MC-glucoside, LC–MS analyses were performed using the whole mixture. MS signals corresponding to the peaks of products were obtained. Ions of m/z 324 and 322 (expulsion of 2H^+^) (Figure S4A), m/z 284 (Figure S4B), and m/z 286 (Figure S4C) indicate the presence of the 3,4-DCA-1-N-glucoside, 2,6-DMP-1-O-glucoside, and 3-MC-1-O-glucoside, respectively. The other ion series in the MS figures were back ground ions. The presence of these unexpected ions could be due to the low concentration of products in the mixture. The UGT activities of the enzyme toward 0.2 mM 3,4-DCA and PCs are shown in Table 1. The enzyme showed the highest UGT activity toward 2,4,5-TCP among the seven substrates tested.

**Table 1:** HPLC analyses of UGT activities of protein extracts from *Pichia pastoris* and *Arabidopsis* plants.

	*Pichia pastoris*		*Arabidopsis thaliana*			
	CK	UGT	WT	UGT-1	UGT-2	UGT-3
2,4,5-TCP	0	230.55±15.09	7.30±0.60	10.95±0.83**	10.50±0.82**	9.06±0.74*
2,6-DMP	0	96.24±8.24	1.17±0.14	1.61±0.12**	1.58±0.13**	1.49±0.11*
phenol	0	104.29±8.17	4.28±0.34	8.84±0.72**	7.82±0.62**	6.53±0.57**
hydroquinone	0	71.16±8.67	7.06±0.63	11.59±0.94**	10.49±0.85**	9.14±0.76*
catechol	6.52±0.53	130.36±14.39	5.65±0.54	9.33±0.86**	7.90±0.58**	6.81±0.57
3-MC	0	23.67±2.28	0.85±0.06	0.92±0.06	1.02±0.08*	0.98±0.07
3,4-DCA	0	68.64±4.06	4.37±0.29	5.45±0.45*	6.06±0.40**	5.04±0.38

Data represent mean ± SD (n = 3). Statistical analyses of differences in VT *Arabidopsis* plants with respect to WT *Arabidopsis* plants and *P. pastoris* expressing *VvUGT72B1* with respect to *P. pastoris* transformed with pPIC9K are performed using Dunnett’s two-tailed *t*-test. Significant difference is denoted with one (P<0.05) or (P<0.01) two asterisks.

### Resistance to 3,4-DCA and PCs in *P. pastoris*

**Figure 4:**
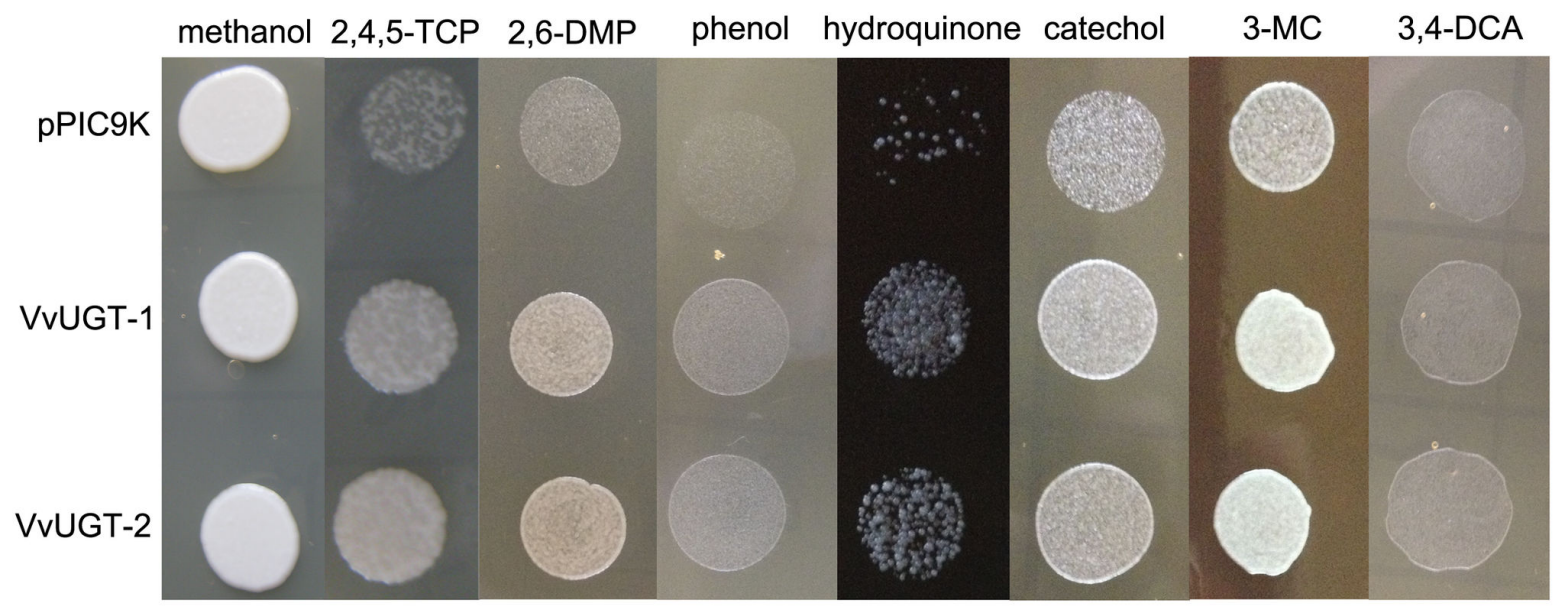
HPLC analyses of xenobiotics and their gluconjugates. The reaction mixes contained protein and UDPG with incubated (A) 2,4,5-TCP, (B) 2,6-DMP, (C) phenol, (D) hydroquinone, (E) catechol, (F) 3-MC, or (G) 3,4-DCA. Protein extracts were from *P. pastoris* expressing VvUGT72B1 (red) or containing empty vector (blue).

3,4-DCA and PCs are highly toxic to *P. pastoris*. Accordingly, we spotted two lines of yeast expressing *VvUGT72B1* and yeast transformed with control pPIC9K plasmid on plates containing xenobiotic. Glucosylation of xenobiotic substances is considered to be part of the phase-II detoxification process in plants. Therefore, overexpression of VvUGT72B1 in *P. pastoris* may enhance its resistance to xenobiotic substances. As expected, VvUGT72B1 yeasts exhibited significantly less growth inhibition than yeast transformed with control pPIC9K plasmid in plates containing 0.46 mM 3,4-DCA, 45.58 µM 2,4,5-TCP, 4.09 mM 2,6-DMP, 8.50 mM phenol, 0.91 mM hydroquinone, 6.36 mM catechol, or 1.21 mM 3-MC (Figure 4).

### Stable expression of VvUGT72B1 in transgenic *Arabidopsis* plants

**Figure 5:**
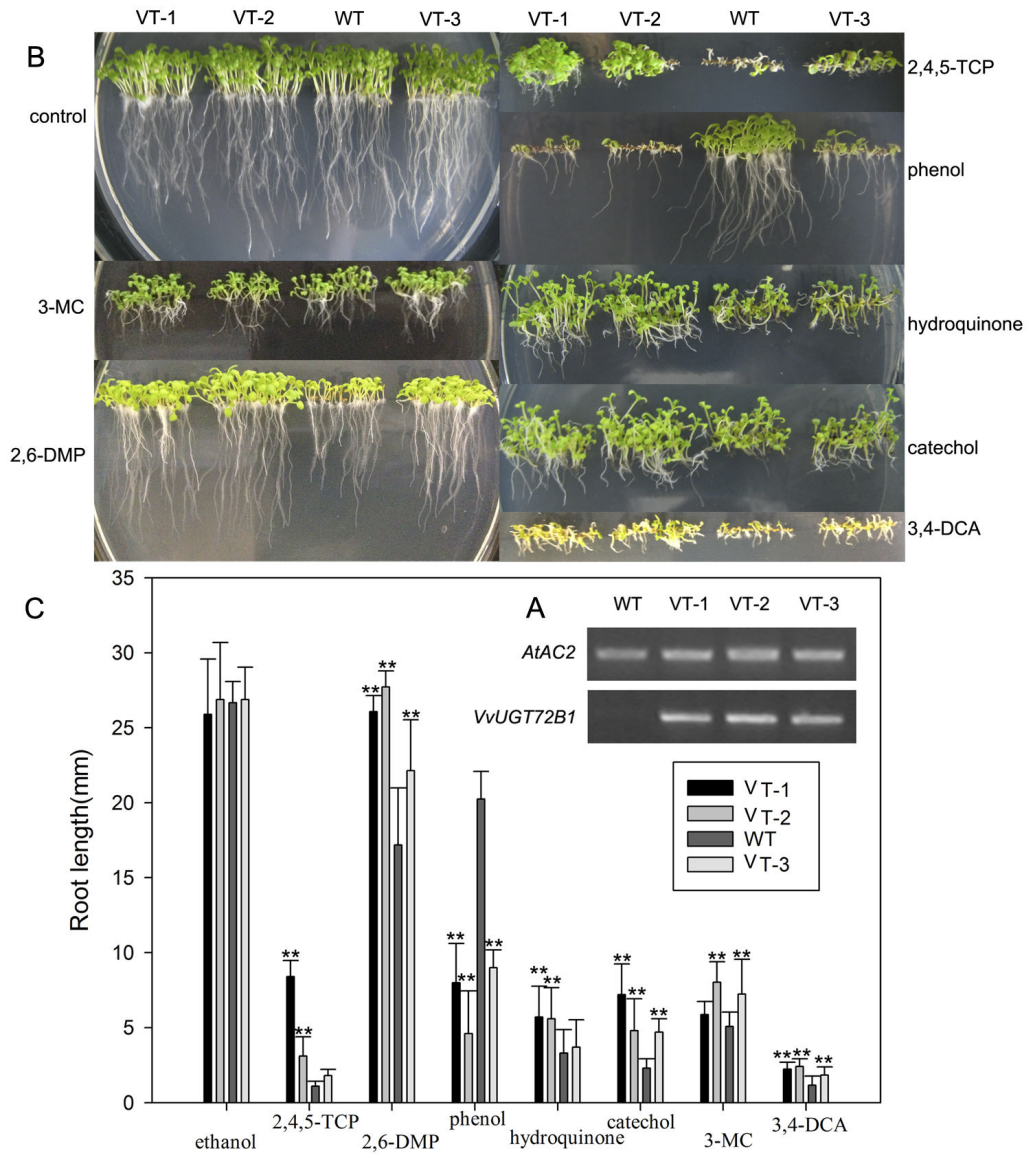
VvUGT72B1 expression in *Arabidopsis* plants. (A) RT-PCR-amplified *VvUGT72B1* fragments from the plants of WT and VT lines (VT-1, VT-2, and VT-3) with *AtAC2* as a reference. (B) Tolerance of *Arabidopsis* plants on plates. WT and VT plants germinated and grown vertically for 10 days on MS agar plates containing methanol, 2,4,5-TCP, 2,6-DMP, phenol, hydroquinone, catechol, 3-MC, or 3,4-DCA. (C) Root length of plants grown on MS agar plates containing PCs or 3,4-DCA. The data are presented as mean ±SD (n = 30). Statistical analyses of differences in VT lines with respect to WT plants is performed using Dunnett’s two-tailed *t*-test. Significant difference is denoted with one (P<0.05) or two (P<0.01) asterisks.

Three homozygous transgenic lines expressing *VvUGT72B1* gene were selected from T_3_ plants using hygromycin selection and RT-PCR. An RT-PCR product of approximately 580 bp of the coding sequence was detected in all three VT lines analyzed, whereas no such signal was detected in WT plants. With *AtAc2* as a reference, VT-1 and VT-2 showed higher expression levels than plant line VT-3 (Figure 5A).

### UGT activities in *Arabidopsis* plants

The glucosyltransferase activities in the VT and WT lines were assayed with crude enzyme extracts from 4-week-old seedlings. All three VT lines showed higher UGTs activities than WT plants during incubation (Table 1).

### Tolerance of homozygous VT plants to xenobiotic on plates

About 30 seeds from each VT and WT lines were sown on MS agar plates containing various concentrations of xenobiotics for 2 weeks. The status and root length of *Arabidopsis* plants are shown in Figures 5B and 5C, respectively. On the control media without xenobiotics, both WT and VT plants appeared equally healthy. On the plates containing 0.20 mM 2,6-DMP, 0.45 mM hydroquinone, 0.18 mM catechol, or 0.40 mM 3-MC, VT lines showed higher tolerance, with broader leaves and longer roots. Although the growth of both WT and VT plants were inhibited, they grew on the plates. At 0.25 mM 3,4-DCA, most seeds of WT line failed to germinate, whereas most seeds of VT lines germinated and produced broader leaves and longer roots. At 30.39 µM 2,4,5-TCP, WT seedlings failed to survive with leaves bleaching, whereas most VT seedlings produced green leaves and longer roots, especially the VT-2 line, although both WT and VT plants were severely inhibited. At 0.40 mM 3-MC, VT lines showed longer roots than WT seedlings. Meanwhile, on the plates containing 0.37 mM phenol, WT seedlings grew healthy, whereas most seeds of VT lines failed to produce shoot. VT lines showed weakened resistance to phenol than WT plants.

### Higher removal efficiency of VT plants toward 3,4-DCA and PCs

Two-week-old WT and VT plants were severely retarded and bleached after culturing in media containing 3,4-DCA. WT plants failed to survive on day 3, whereas VT plants were still alive. As shown in Figure S5, the concentrations of 3,4-DCA in the media with VT plants degraded faster than that in the media with WT plants. 3,4-DCA-glucoside was observed in media cultured with plants. The concentrations of 3,4-DCA-glucoside in media with VT plants were higher than that in media with WT plants, whereas no 3,4-DCA-glucoside was observed in media without plants. VT plants uptook more 3,4-DCA and exported more 3,4-DCA-glucoside to the media than WT plants. The 3,4-DCA concentrations increased in media without plants for 3 days.

The PCs concentrations changed (increased or decreased) in media without plants. However, 2,4,5-TCP-glucoside, PGPS, arbutin, catechol-glucoside, 2,6-DMP-glucoside, or 3-MC-glucoside were not observed in these media. PCs in the media were toxic but not lethal to WT and VT plants. The degradation efficiencies of PCs in the media cultured with VT plants were higher than those in media with WT plants. PGPS, 2,6-DMP-glucoside, and 3-MC-glucoside were observed in media cultured with plants, whereas VT plants more efficiently exported these glucosides than WT plants.

**Figure 6:**
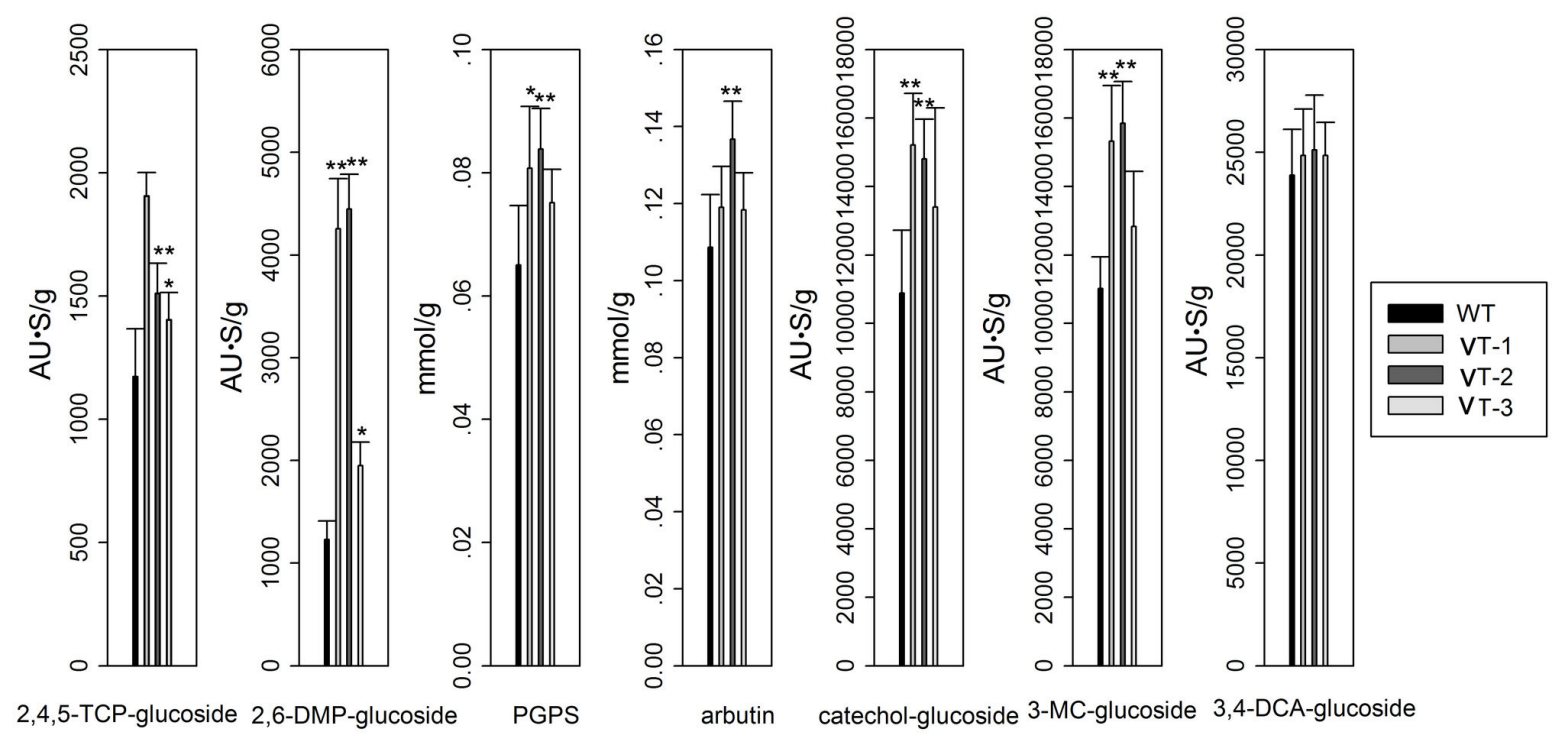
HPLC analyses of plant extracts. The contents of glucosides of plants were determined after the 2-week-old WT and VT lines were cultured in MS liquid medium containing 2,4,5-TCP, 2,6-DMP, phenol, hydroquinone, catechol 3-MC, or 3,4-DCA for 1 day. Values represent the mean of triplicate incubations with error bars showing SD (*n* = 3). Statistical analysis of differences in VT lines with respect to WT plants in each treatment is performed using Dunnett’s two-tailed *t*-test. Significant difference is denoted with one (P <0.05) asterisk.

The contents of 3,4-DCA-glucoside, 2,4,5-TCP-glucoside, PGPS, arbutin, catechol-glucoside, 2,6-DMP-glucoside, or 3-MC-glucoside synthesized in the plants were also determined after 1 day. VT plants accumulated more 3,4-DCA-glucoside, 2,4,5-TCP-glucoside, PGPS, arbutin, catechol-glucoside, 2,6-DMP-glucoside, or 3-MC-glucoside than WT plants (Figure 6).

## Discussion

The glucosylation of phytotoxic and xenobiotic substances is considered to be part of the phase-II detoxification process, which enables plants to cope with the enormous diversity of toxic microbial metabolites [10,27,28]. Then, conjugated xenobiotics and phytotoxic are compartmentalized in vacuoles, bound to cell wall components such as lignin or hemicellulose in phase III [28,29], or exported to surrounding media as previously described [14,30]. PCs and 3,4-DCA are suitable for glucosylation because of hydroxyl or amino groups in the benzene ring.

Many plant UGTs activities toward xenobiotics are region and substrate specific. Consequently, a few kinds of xenobiotics have been determined in previous individual studies [13,14,17,18,30]. In current work, enhanced glucosylation and remediation of six kinds of phenolic compunds and 3,4-dichloroaniline by *Arabidopsis* plants expressing VvUGT72B1 were confirmed. Our findings provide a broader spectrum remediation strategy for the phytoremoval and degradation of phenolic compounds and 3,4-dichloroaniline than previous works [13,14,18,30]. As 1-O-glucosides of these six PCs and 1-N-glucoside of 3,4-DCA were produced in the mixture by VvUGT72B1. We also can consider VvUGT72B1 to be both *O*-glucosyltransferase and *N*-glucosyltransferase.

The approach used to screen *P. pastoris* expressing VvUGT72B1 was efficient. The synthesized *VvUGT72B1* gene was highly expressed in *P. pastoris*. The result of resistance test indicated that VvUGT72B1 was able to protect *P. pastoris* against the six PCs and 3,4-DCA. Given that no UGT activity toward PCs and 3,4-DCA was observed in the control, recombinant VvUGT72B1 protein reached almost 50% of the total protein and accounted for 100% of UGT activities in *P. pastoris*, except for catechol. VvUGT72B1 showed much higher expression level in *P. pastoris* than that in *Arabidopsis* plants by comparing either UGT activities or recombinant VvUGT72B1 protein content.

In this study, we also found that VT *Arabidopsis* plants showed higher UGTs activities WT plants and VvUGT72B1 enhanced the removal efficiencies of *Arabidopsis* plants for all tested xenobiotics. The processing and subsequent intracellular transport of glycosidic conjugates of xenobiotics in plants was species dependent [30]. 3,4-DCA-glucoside and PGPS formed in *A. thaliana* can be transported to the surrounding media [14,30]. In the present study, we initially proved that 2,6-DMP-glucoside and 3-MC-glucoside formed in *A. thaliana* were transported to the surrounding media. *Arabidopsis* plants expressing VvUGT72B1 exported more 3,4-DCA-glucoside, PGPS, 2,6-DMP-glucoside, and 3-MC-glucoside to the surrounding media than WT plants. VvUGT72B1 enhanced the removal efficiencies of *Arabidopsis* plants for all tested xenobiotics. The glucosides of hydroquinone, catechol, and 2,4,5-TCP in the plants may either be gradually degraded further by endogenous enzyme or entirely sequestered within plants in a form that may not be easily extractable, and further confirmation is required.

3,4-DCA, 2,4,5-TCP, phenol, hydroquinone, and catechol had cellular toxicities to *Arabidopsis*, as previously reported [11,13,14]. In this study, significant toxic effects of 2,6-DMP and 3-MC to *Arabidopsis* were demonstrated. The result of resistance tests indicated that VvUGT72B1 was able to protect *Arabidopsis* against all xenobiotics tested except for phenol. VT plants in plates displayed lower resistance to phenol than WT plants probably due to the failure of germinating VT seeds in expressing adequate exporter to reduce overproduced PGPS, which had higher intracellular toxicity to *Arabidopsis* plants than phenol as previously described [14].

Future studies concentrated on the roles of VvUGT72B1 in plants widely used for bioremediation. Transgenic plants expressing *VvUGT72B1* gene may provide a generally applicable strategy for bioremediation of various PCs and 3,4-DCA from soil and water.

## Supporting Information

Figure S1 **Nucleotide sequence of sense strand alignment of the synthesized and wild-type VvUGT72B1 gene.** “S” represents the synthesized *VvUGT72B1* gene, and “WT” represents the wild-type *VvUGT72B1* gene. The unmodified nucleotides of the synthesized gene are represented as ‘·’.
(TIF)

Figure S2 **Structures of xenobiotics undergoing conjugation.** (1) 2,4,5-TCP, (2) 2,6-DMP, (3) phenol, (4) hydroquinone, (5) catechol, (6) 3-MC, and (7) 3,4-DCA.
(TIF)

Figure S3 **Comparative analyses of the predicted structures of VvUGT72B1 and AtUGT72B1.** (A) Crystal structure showing the 3D folding of elements of the secondary structure with α-helices shown in red and β-strands in cyan. The 3D folding of elements of PSPG motif is shown in blue. (B) Stereo diagram showing the superimposition of the donor binding pockets (red) of PSPG (blue solid ribbon), residues of enclosing TCP pockets (saffron yellow), and residues interacting with acceptor (purple) of VvUGT72B1 and AtUGT72B1. UDP-glucose and TCP are shown as black and green stick models, respectively.
(TIF)

Figure S4 **LC-MS analyses of products.** LC-MS analyses of VvUGT72B1 products after incubation with (A) 3,4-DCA, (B) 2,6-DMP, and (C) 3-MC. The whole mixtures were used for analyses.
(TIF)

Figure S5 **HPLC analyses of the removal efficiency of PCs and 3,4-DCA in media.** The concentrations of 2,4,5-TCP, 2,6-DMP, phenol, hydroquinone, catechol, 3-MC, and 3,4-DCA, as well as the produced 2,6-DMP-glucoside, PGPS, 3-MC-glucoside, and 3,4-DCA-glucoside in the medium were determined after WT and VT lines were cultured in MS liquid medium containing PCs or 3,4-DCA every day. The control was a medium with PCs or 3,4-DCA but no *Arabidopsis* plants. Values are the mean of triplicate incubations with error bars showing SD (*n* = 3).
(TIF)
