# Supplementary figures and images for: Characterization of a Bifunctional O- and N-Glucosyltransferase from Vitis vinifera in Glucosylating Phenolic Compounds and 3,4-dichloroaniline in Pichia pastoris and Arabidopsis thaliana
Source: PLoS One. 2013 Nov 14;8(11):e80449. doi: 10.1371/journal.pone.0080449 (PMC3828253; doi:10.1371/journal.pone.0080449)

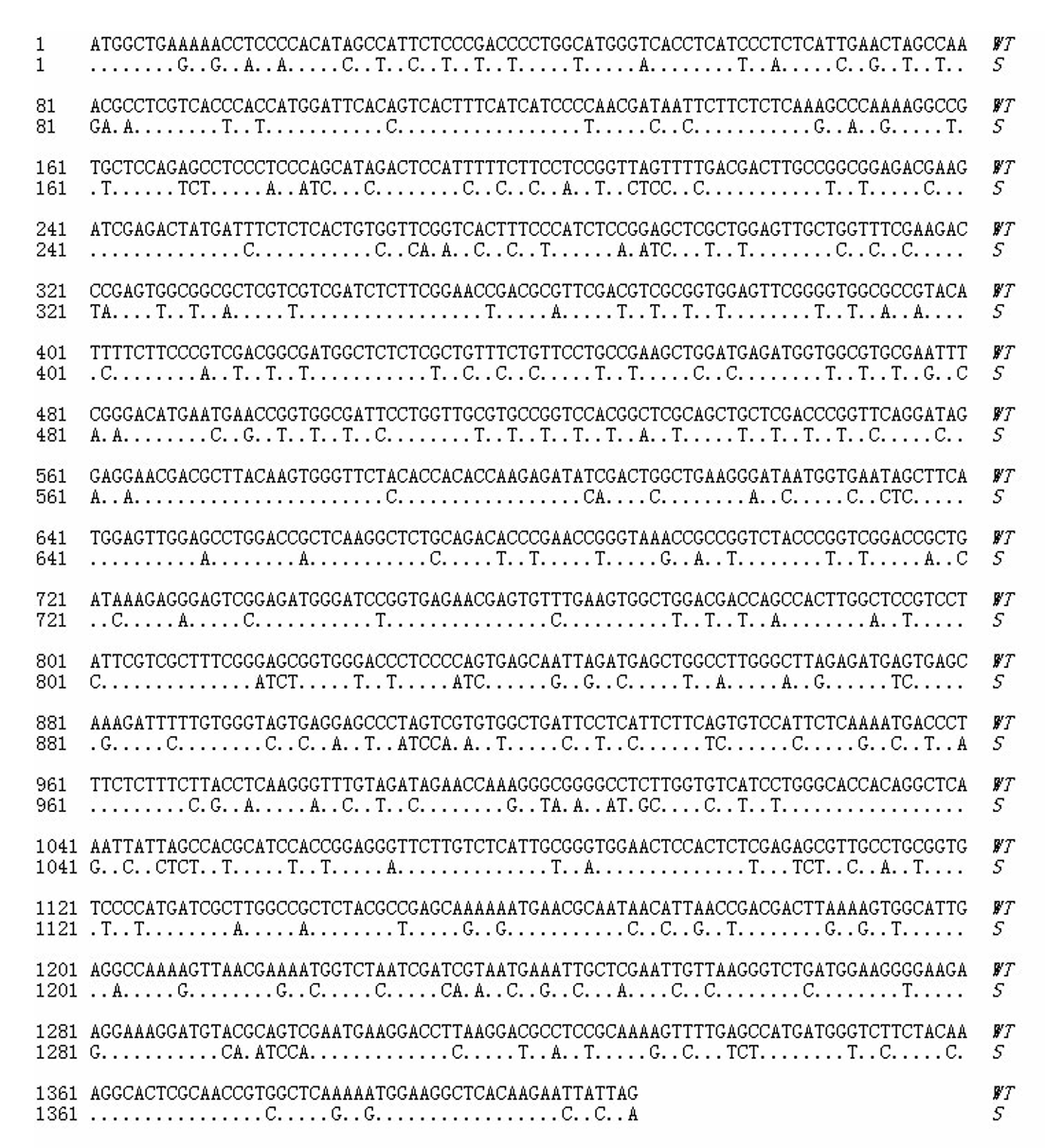

Supplement: Figure S1 — Nucleotide sequence of sense strand alignment of the synthesized and wild-type VvUGT72B1 gene. “S” represents the synthesized VvUGT72B1 gene, and “WT” represents the wild-type VvUGT72B1 gene. The unmodified nucleotides of the synthesized gene are represented as ‘·’. (TIF) [file pone.0080449.s001.tif]

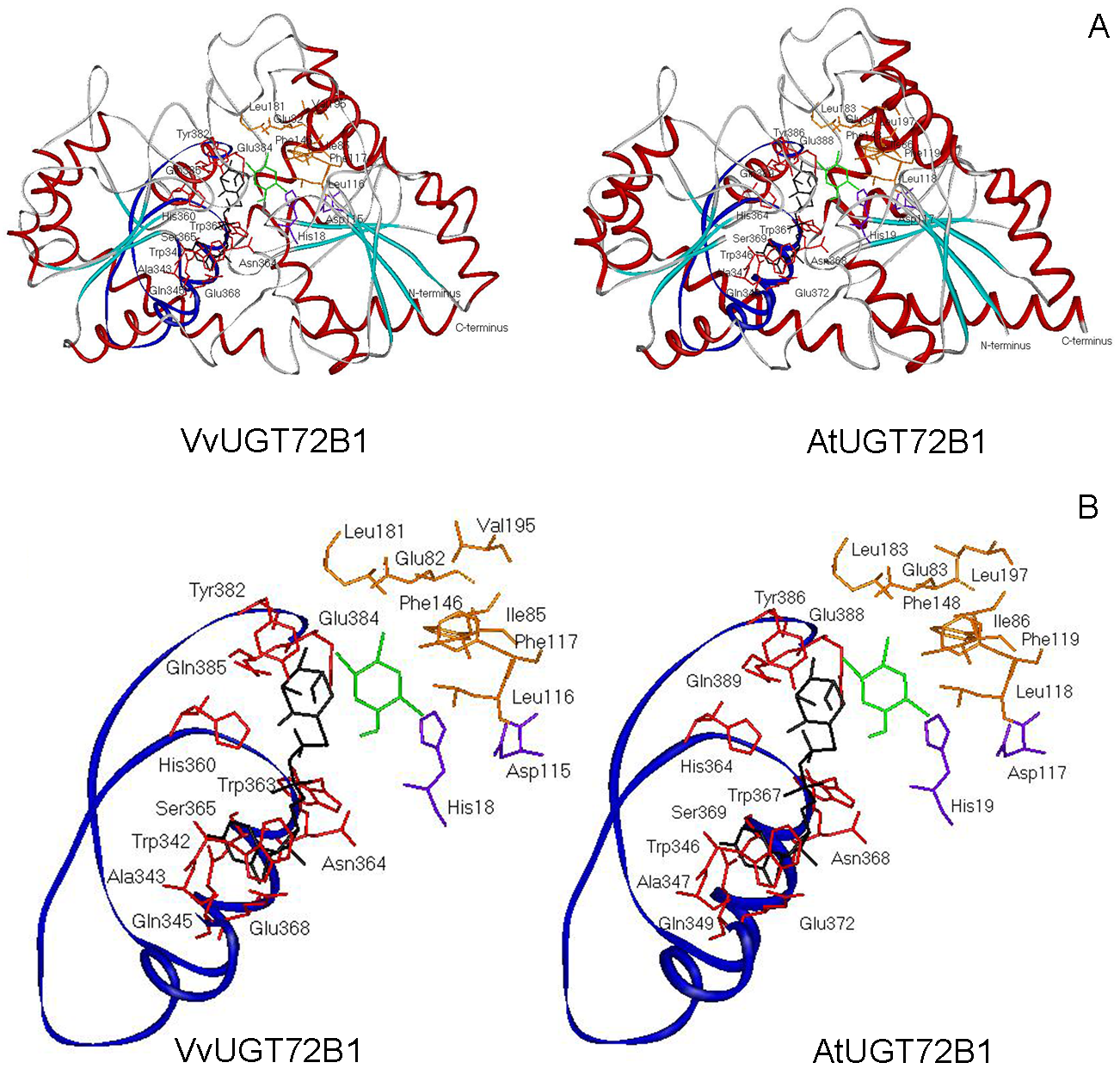

Supplement: Figure S2 — Structures of xenobiotics undergoing conjugation. (1) 2,4,5-TCP, (2) 2,6-DMP, (3) phenol, (4) hydroquinone, (5) catechol, (6) 3-MC, and (7) 3,4-DCA. (TIF) [file pone.0080449.s002.tif]

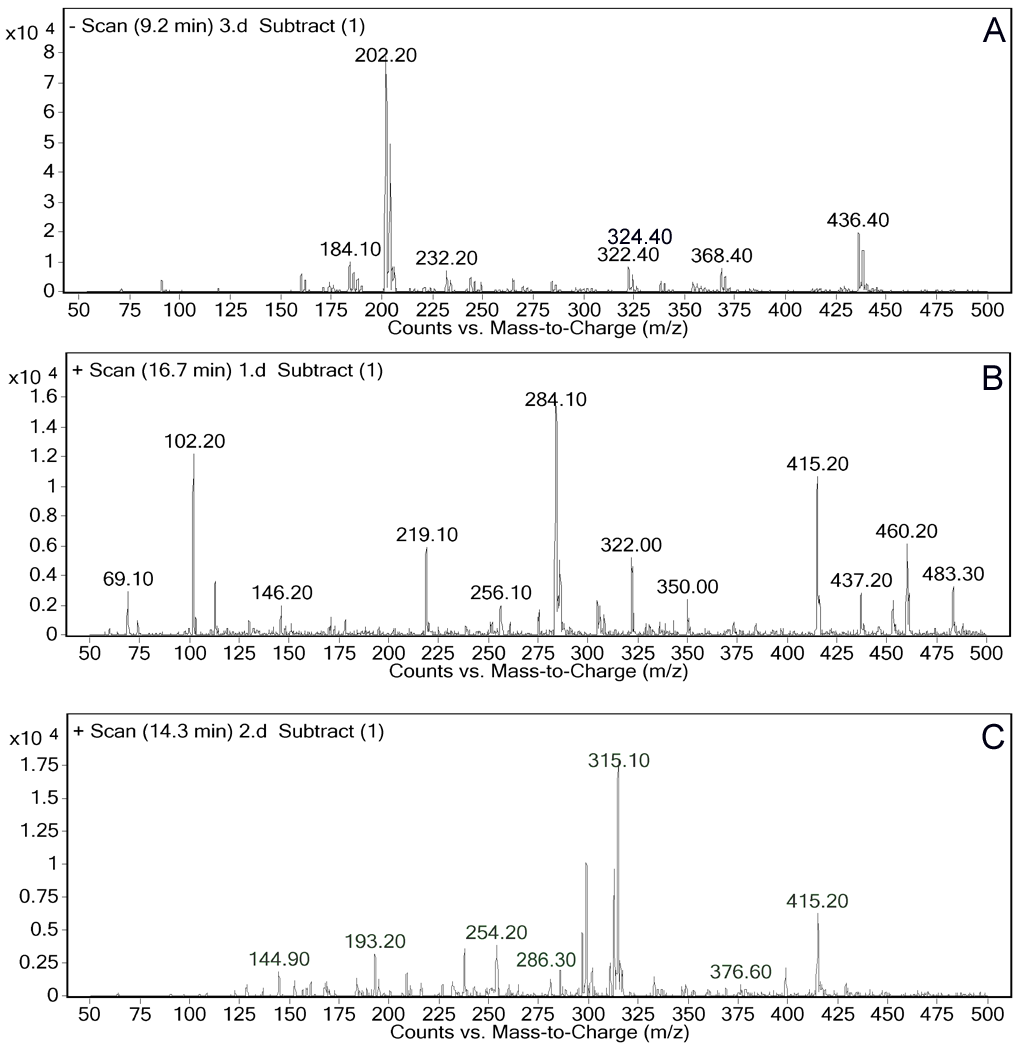

Supplement: Figure S4 — LC-MS analyses of products. LC-MS analyses of VvUGT72B1 products after incubation with (A) 3,4-DCA, (B) 2,6-DMP, and (C) 3-MC. The whole mixtures were used for analyses. (TIF) [file pone.0080449.s004.tif]
